# The Dundee Infirmary

**Published:** 1888-12-08

**Authors:** Angus Fraser

**Affiliations:** Physician to the Aberdeen Royal Infirmary


### THE DUNDEE INFIRMARY.

By Angus Fraser, M.D., Physician to the Aberdeen Royal Infirmary.

In The Hospital for November 17th, 1888, I see a notice of the Dundee Royal Infirmary, which in some respects seems to me so unfair to the management of that institution, that I ask your permission to offer a contradiction to some of the remarks made. I have frequently visited and inspected the Dundee Infirmary within the last few years, and am therefore able to speak from repeated personal observation. The remark to which I specially take objection is that the wards are "dreary, neglected, and repelling." A more unfair and unfounded comment it is impossible to conceive. When one speaks of wards as being "neglected," it either means that they are dirty and untidy, or that there is inefficient supervision in the way of nursing. Now in Dundee the wards are as clean and tidy as fresh paint and constant scrubbing can make them. The walls are bright and tastefully painted, the beds and bedding scrupulously clean and comfortable-looking, and the floors correspond. In fact, as a lady (very capable of judging, and who visited the hospital on my recommendation) said, "I never saw any private house or public institution in a state of such absolute cleanliness." And as to nursing, there is an ample well-trained staff; and I never saw a ward in any of my numerous visits in which there was not one or more nurses attending on the patients. Anyone who takes an interest in hospitals knows that wards may look clean, and yet the institution may not be well kept. It is in the bath-rooms, lavatories, w.c.'s, and duty-rooms, that one looks for defects in the way of cleanliness; but in the Dundee Infirmary these places also stand the most minute inspection. It is true that, on sanitary grounds, Dr. M'Cosh, the superintendent, objects to pictures on the walls, so that these are not to be found in Dundee; but, although I do not agree with him on this point, I must confess that to my mind the brightly and freshly painted wards in Dundee are far preferable to the dingy-looking walls, studded higgledy-piggledy with tawdry chromographs and high-coloured scripture texts, which one sees in so many hospitals.

Aberdeen, Dec. 1st, 1888.

